# Supplementary material for: Anti-Myocardial Infarction Effects of Radix Aconiti Lateralis Preparata Extracts and Their Influence on Small Molecules in the Heart Using Matrix-Assisted Laser Desorption/Ionization–Mass Spectrometry Imaging
Source: Int J Mol Sci. 2019 Sep 29;20(19):4837. doi: 10.3390/ijms20194837 (PMC6801437; doi:10.3390/ijms20194837)
Supplement: Supplementary file 1 [file ijms-20-04837-s001.pdf]

International Journal of *Molecular Sciences*

MDPI

Supplementary Material

Anti-myocardial Infarction Effects of Radix Aconiti Lateralis Preparata extracts and their Influence on Small Molecules in the Heart using Matrix-assisted Laser Desorption/Ionization–Mass Spectrometry Imaging

Hao Wu [1,2], Xi Liu [1,2], Ze-yu Gao [1,2], Zhen-feng Dai [1,2], Ming Lin [1,2], Fang Tian [1,2], Xin Zhao [1,2], Yi Sun [1,2] and Xiao-Ping Pu [1,2,*]

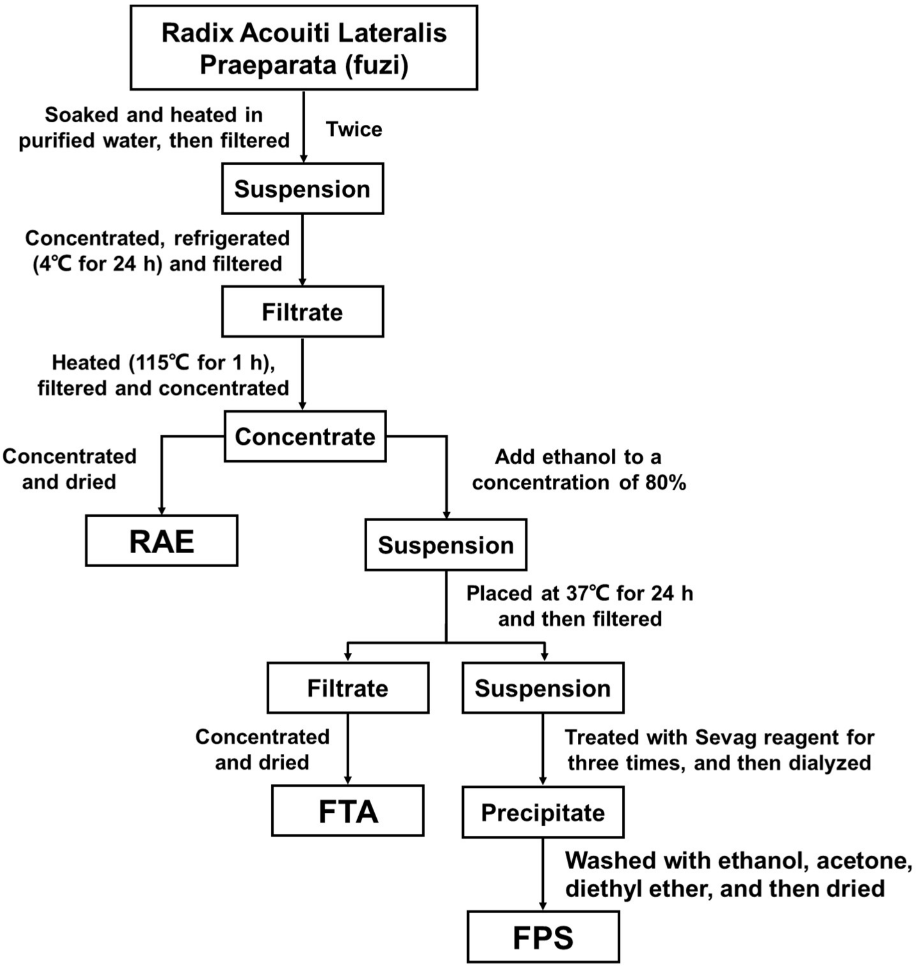

Supplementary Figure S1. Work flow of the Radix Aconiti Lateralis Preparata processing

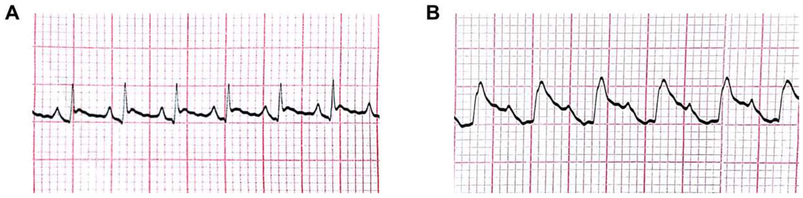

Supplementary Figure S2. Electrocardiograms of rats. (A) Normal electrocardiogram (50 mm/s; 10 mm/mV). (B) Myocardial infarction electrocardiogram (50 mm/s; 10 mm/mV).

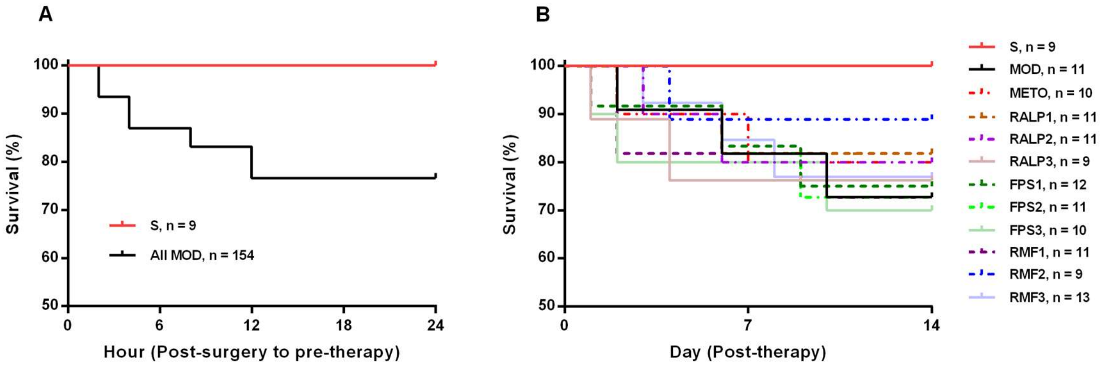

Supplementary Figure S3. Effect of RAE, FPS, and FTA on the survival of the rats with myocardial infarction. (A) Post-surgery to pre-therapy survival curve. (B) Post-therapy survival curve. S, sham surgery group; MOD, model group; METO, metoprolol-treated group; RAE1/2/3, 1.6/0.8/0.4 g/kg Radix Aconiti Lateralis Preparata extract groups; FPS1/2/3, 1.6/0.8/0.4 g/kg fuzi polysaccharides groups; FTA1/2/3, 1.6/0.8/0.4 g/kg fuzi total alkaloid groups.

Supplementary Table S1. Animal grouping and extracts administration

Groups	Drug treatment	Administration dose (g/kg)	Total numbers of rats	Survival numbers of rats before sacrifice
**S**	N.S.[1]	10 mL/kg	9	9
**MOD**	N.S.	10 mL/kg	11	8
**METO**	Metoprolol	1.5 mg/kg	10	8
**RAE1**	RAE[2]	1.6	11	9
**RAE2**	RAE	0.8	11	9
**RAE3**	RAE	0.4	9	7
**FPS1**	FPS[3]	1.6	12	9
**FPS2**	FPS	0.8	11	8
**FPS3**	FPS	0.4	10	7
**FTA1**	FTA[4]	1.6	11	8
**FTA2**	FTA	0.8	9	8
**FTA3**	FTA	0.4	13	10

[1] N.S., normal saline.

[2] RAE, radix Aconiti Lateralis Preparata extract.

[3] FPS, fuzi polysaccharides.

[4] FTA, fuzi total alkaloid.
